# Breast cancer genetic counseling among Dutch patients from Turkish and Moroccan descent: participation determinants and perspectives of patients and healthcare professionals

**DOI:** 10.1007/s12687-016-0290-4

**Published:** 2017-01-12

**Authors:** J.E. Baars, A.M. van Dulmen, M.E. Velthuizen, E. van Riel, M.G.E.M. Ausems

**Affiliations:** 1 Department of Genetics, Division of Biomedical Genetics, University Medical Center Utrecht, PO Box 85090, 3508 AB Utrecht, the Netherlands; 2 NIVEL (Netherlands institute for health services research), Utrecht, the Netherlands; 3 Department of Primary and Community Care, Radboud University Medical Center, Nijmegen, the Netherlands; 4 Faculty of Health Sciences, University College of Southeast Norway, Drammen, Norway

**Keywords:** Breast cancer, Ethnic minority, Genetic counseling, Referral

## Abstract

Lower participation rates in cancer genetic counseling are observed among different ethnic minorities. The goal of our study is to gain insight into determinants of Turkish and Moroccan patients’ participation in breast cancer genetic counseling and DNA testing, from the point of view of healthcare professionals and patients. Questionnaire-based telephone interviews about awareness, perceptions, and reasons for (non-) participation in cancer genetic counseling were conducted with 78 Dutch breast cancer patients from Turkish and Moroccan descent. The interviews were held in Arabic, Berber, Turkish, or Dutch by bilingual research assistants. Additionally, 14 breast cancer patients participated in one of two focus group meetings, and two focus groups were held with 11 healthcare professionals. SPSS and QSR Nvivo were used to examine the quantitative and qualitative data, respectively. Half of the total group of patients (*N* = 78) and 79% of patients eligible for genetic counseling and testing (*N* = 33) were aware of the possibility of genetic counseling. The most important determinants for nonparticipation in genetic counseling were experienced difficulties in patient-doctor communication, cultural factors (e.g., social norms), limited health literacy, limited knowledge of the family cancer history, and anxiety about cancer. Religious beliefs and knowing personal and family members’ breast cancer risks were motives to obtain genetic counseling. Despite the fact that our study showed that Moroccan and Turkish women reported several personal motives to obtain genetic counseling and testing (GCT), patients and healthcare professionals experience significant language and health literacy difficulties, which make it harder to fully access health care such as genetic counseling and testing.

## Introduction

Breast cancer is the most frequently occurring cancer among women worldwide. Breast cancer patients with a young age at diagnosis (<40 years) or a family history of breast or ovarian cancer have an increased risk of carrying a BRCA 1/2 gene mutation. Despite a steadily growing interest in breast cancer genetic counseling and DNA testing in the past decade, diverse ethnic minority groups seem to be underrepresented within the family cancer clinics (Armstrong et al. 2003; Armstrong et al. 2005; Forman and Hall 2009; Levy et al. 2011; Sussner et al. 2009; Sussner et al. 2011; van Riel et al. 2012). Recently, we showed in a registry-based study in the Netherlands that Turkish and Moroccan ethnicity was associated with a lower participation rate in breast cancer genetic counseling and testing (GCT), mainly due to a lower referral rate among patients who were younger than 40 years of age at diagnosis (Baars et al. 2016). This suggests that migrant Turkish and Moroccan breast cancer patients, as well as their family members, are less likely to benefit from the health benefits of GCT because information about increased cancer risks and screening possibilities to facilitate early diagnosis might not reach them.

In the Netherlands, Turks and Moroccans belong to the largest migrant groups, with nearly 400,000 inhabitants with a Turkish background and 375,000 with a Moroccan background (CBS 2014). Higher relative excess breast cancer mortality in Turkish and Moroccan women points towards possible inadequate access to health care and treatment, especially the participation in screening programs (Arnold et al. 2013). Religious and cultural beliefs seem to influence the utilization of cancer screening (Rajaram and Rashidi 1999; Rashidi and Rajaram 2000; Matin and LeBaron 2004; Hasnain et al. 2011), as well as language (Topal et al. 2015) which is also a known factor to lead to lower participation rates in GCT in general (Forman and Hall 2009). To our knowledge, however, determinants of participation in GCT are not known for Moroccan and Turkish women, specifically. Because of the indications that these migrant groups are less likely to be reached, our aim is to study determinants of GCT uptake among Turkish and Moroccan breast cancer patients.

As with other health interactions, referrals to GCT need to be a shared decision making (Smets et al. 2007). To fully understand the perceptions of both patients and healthcare professionals on the participation in GCT, we will take into account the broader context, that is, the breast cancer patient’s experiences with health care as provided by their surgeon and the communication about cancer in the family. Our research questions were as follows: *What is the awareness about breast cancer GCT among Moroccan and Turkish breast cancer patients and what are possible determinants of GCT uptake among Turkish and Moroccan breast cancer patients from the point of view of healthcare professionals and patients?*

## Methods

### Study population

The study included Moroccan and Turkish patients who had been diagnosed with breast cancer between January 2007 and December 2012 in one of six hospitals in the Utrecht region and in Amsterdam, the Netherlands.

### Procedure

Turkish and Moroccan patients that were identified according to a name-based approach (Baars et al. 2016; Razum et al. 2001; Spallek et al. 2006, 2009; Hoopman et al. 2009) and who had been diagnosed with breast cancer (*n* = 145) were invited to take part in a study that investigated health care for Turkish and Moroccan breast cancer patients. To minimize possible selection bias, genetic counseling was not mentioned in the recruitment letter. This letter was written in Turkish and Dutch for the Turkish patients and in Standard Arabic and Dutch for the Moroccan patients. A research team of four bilingual research assistants approached and interviewed the respondents. The research assistants were trained in how to administer the questionnaire and when to ask further. The interviews were held between November 2013 and March 2014 in four languages (Arabic, Berber, Turkish, and Dutch). Non-response included *n* = 19 patients who could not be reached by telephone, *n* = 16 patients who did not want to be reminded of the period of having cancer and refrained from participation, *n* = 6 patients who were unable to participate due to illness or age, *n* = 2 did not have time, *n* = 10 reported various other reasons, and *n* = 14 patients who did not give a reason why they did not want to participate.

Respondents were generally younger than non-respondents (mean age at the start of the interviews 49.41 (SD 9.46) versus 54.56 (SD 12.82); *p* = 0.008)). In total, 78 patients (54%) were interviewed. Additionally, in October 2014, a sample of seven of these patients participated in a focus group with Moroccan patients. In the same month, a focus group of seven Turkish patients was organized of whom five were recruited through mediation of an organization for migrant breast cancer patients and two who had previously participated in our study. The focus group interview among Moroccan women was conducted mainly in the Dutch language, and the Turkish group was conducted in the Turkish language. In these focus groups, possible determinants of migrants’ participation in breast cancer GCT were further explored within the context of the cultural and religious beliefs within these populations. Possible determinants were also discussed within two focus groups of healthcare professionals: a focus group of seven physicians (surgical oncologists and a radiation oncologist) and a group of four nurse practitioners from different hospitals that were held in November and December 2014.

### Measures

The telephone interviews were structured using closed and open questions. Closed questions, with a number of reply options to be filled in by the interviewer, were used to inquire about the patients’ socio-demographic characteristics (marital status, urban or rural family origin, educational level, employment status, religion) based on Hosper (2007); level of acculturation (most common spoken language, most common language of internal monolog, ethnic identity and birthplace) based on Gul et al. and Coronado et al. (Gul and Kolb 2009; Coronado et al. 2005); family-related issues (family history of breast and ovarian cancer, family communication about breast cancer (open question)); and health care (patient satisfaction with received care and information, communication with their surgeon). Open questions focusing on possible risk factors for breast cancer included the role of religion during periods of illness, awareness of breast cancer GCT, knowledge about breast cancer GCT, referral to GCT, and motivators and barriers related to breast cancer GCT. During focus group meetings, several factors that were retrieved from the interviews and could be related to participation in GCT (family communication about breast cancer, communication with the physician, religious point of view on GCT, anxiety about breast cancer GCT, and other factors) were reflected upon within the group (Ruff et al. 2005).

### Statistical analysis

Descriptive statistics derived from the interviews, tests to identify differences between groups (χ^2^ tests and *t* tests), and logistic regression analyses were performed with SPSS. The focus groups were audiotaped and transcribed verbatim. Data resulting from the open questions and the focus groups were thematically analyzed by researchers JB and EvR (Ruff et al. 2005; Braun and Clarke 2006) with the use of QSR Nvivo 10.

During thematic analysis, themes and patterns within the qualitative data were identified, described, and analyzed (Braun and Clarke 2006). In addition, an inductive approach was used in which we acknowledged the ways individuals make meaning of their experience (Braun and Clarke 2006). The open questions from the first ten interviews and all four focus groups were coded by two researchers (JB and EvR) after which they created a unified coding scheme. Subsequent interviews were compared with these existing codes to identify similarities and differences. The codes were grouped into themes and conceptual categories that were discussed within the research team.

## Results

### Socio-demographic variables

Socio-demographic variables of the 78 patients that were interviewed are shown in Table 1. The Moroccan patients who participated were, on average, 6 years younger than the Turkish patients (*p* = 0.004). Nearly all women were born in Morocco or Turkey. Of all the women, both of their parents were born in Morocco (*n* = 52) or Turkey (*n* = 26) (not shown in table). Most women (64%; *n* = 50) originated from villages, 6% (*n* = 5) of the women had either a mother or father who lived in a village, and 30% (*n* = 23) reported that both their parents had lived in a city (not shown in table).

**Table 1:** Socio-demographic characteristics

	Moroccan *N* = 52	Turkish *N* = 26	Total *N* = 78	*p* value
Age at diagnosis^a^	44.22 (7.99)	50.51 (10.26)	46.32 (9.24)	0.004
Educational level^b^				
None	35% (18)	27% (7)	32% (25)	-
Primary school	15% (8)	54% (14)	28% (22)	
Low	19% (10)	0 (0)	13% (10)	
Middle	23% (12)	11% (3)	19% (15)	
High	8% (4)	8% (2)	8% (6)	
Daytime activity				
Paid job	13% (7)	15% (4)	14% (11)	-
Disabled, unemployed	25% (13)	42% (11)	31% (24)	
Housewife	62% (32)	39% (10)	54% (42)	
Retired	0% (0)	4% (1)	1%(1)	
Religion				
Islam	100% (52)	92% (24)	97% (76)	NS
None	0% (0)	8% (2)	3% (2)	
Country of birth				
Morocco	96% (50)		64% (50)	-
Turkey		92% (24)	31% (24)	
Netherlands	4% (2)	8% (2)	5% (4)	
Acculturation				
Speak mostly T/M	67% (35)	77% (20)	71% (55)	NS
Think mostly T/M	69% (35)	85% (22)	74% (57)	NS
Feel mostly T/M	73% (38)	54% (14)	67% (52)	NS
Family characteristics				
Presence of				
Sisters	98% (51)	96% (25)	97% (76)	NS
Children	96% (50)	96% (25)	96% (75)	
Daughters	75% (39)	77% (20)	76% (59)	
Age of youngest child^c^				
0–18 years	76% (38)	32% (8)	61% (46)	0.0001
≥18 years	24% (12)	68% (17)	39% (29)	
Relatives with BC				
First degree	10% (5)	19% (5)	13% (10)	NS
Do not know	2% (1)	(0)	1% (1)	
Second degree	19% (10)	12% (3)	17% (13)	
Do not know	8% (4)	4% (1)	6% (5)	
Relatives with OC				
First degree	0 (0)	4% (1)	1% (1)	-
Second degree				
Yes	2% (1)	0 (0)	1%(1)	-
Do not know	18% (9)	4% (1)	13% (11)	
Family communication BC				
Relatives are aware of patient’s BC	62% (32)	92% (24)	72% (56)	0.004

*-* no χ^2^ could be calculated as the conditions of this test could not be fulfilled (>20% of the cells has an expected count <5), *T/M* Turkish/Moroccan, *BC* breast cancer, *OC* ovarian cancer, *NS* not significant

^a^mean (SD)

^b^ *Low* lower secondary or second stage of basic education, *medium* (upper) secondary education, *high* tertiary education

^﻿c^of those with children﻿﻿

A large proportion of the Moroccan women (76%) had one or more children under 18 years of age, and this was higher than among the Turkish women (32%) (see Table 1). The self-reported data on family cancer history are presented in Table 1.

#### Awareness of breast cancer GCT

Genetic and familial factors were mentioned by 22% of the 78 patients as a possible cause of breast cancer (open question). Other perceived risk factors for breast cancer were distress and grief (54%) which could lower the immune system and could cause breast cancer, lifestyle factors (40%), and Allah’s will (21%) (not shown in Table).

When asked directly about breast cancer GCT, about half of the breast cancer patients were aware of this possibility (see Table 2), mainly because their surgeon had informed them while asking about breast cancer in their family. Among Moroccan women in general, awareness about GCT was higher (60%) compared to that in the Turkish women (31%; *p* = 0.016). Univariate analysis among the total group of migrant patients showed that awareness was positively associated with having family members with breast and/or ovarian cancer. A higher proportion of women who reported a first or second degree family member with breast and/or ovarian cancer was aware of breast cancer GCT (80%), compared to women with no family history of breast and/or ovarian cancer (40%; *p* = 0.002). Negative associations were found for age at breast cancer diagnosis. Mean age at diagnosis was 42.10 (SD 7.20) among those who reported to be aware of breast cancer GCT and 50.54 (SD 9.21) among the women who reported they had never heard about breast cancer GCT (*p* = 0.0001). Furthermore, awareness was associated with variables related to acculturation (those who speak mostly Turkish/Moroccan (T/M) versus those who speak as much T/M as Dutch or mostly Dutch (35 versus 87% aware; *p* = 0.0001); think mostly in T/M (32 versus 100%; *p* = 0.0001); feel mostly T/M (37 versus 77%; *p* = 0.001)) and the use of an interpreter. A lower proportion of women whose visits to the doctor had been translated were aware of GCT (34%), as compared to those who did not use an interpreter (77% aware, *p* = 0.0001). When controlled for age at diagnosis in logistic regression analyses, speaking mostly T/M (OR 0.09; CI 0.02–0.40), feeling mostly T/M (OR 0.21; CI 0.06–0.67) and having an interpreter during the doctor visits (OR 0.29; CI 0.09–0.87) remained associated with lower awareness. In all models, age at diagnosis persisted as a significant predictor of awareness of GCT.

**Table 2:** Awareness and uptake of breast cancer GCT

	Moroccan *N* = 52	Turkish *N* = 26	Total *N* = 78	Total patients meeting GCT referral criteria *N* = 33
Aware of GCT				
Yes: % (*N*)	59.6% (31)*	30.8% (8)*	50.0% (39)	78.8% (26)
Through^a^				
Surgeon	51.6% (16)	87.5% (7)	59.0% (23)	65.4% (17)
Oncologist	12.9% (4)	0	10.3% (4)	11.5% (3)
Television/media	9.7% (3)	0	7.7% (3)	7.7% (2)
Friends/family	22.6% (7)	12.5% (1)	20.5% (8)	11.5% (3)
Do not know	3.2% (1)	0	2.6% (1)	3.8% (1)
Uptake GCT	32.6% (17)	26.9% (7)	30.8% (24)	63.6% (21)

*p = 0.016

^a^No χ^2^ could be calculated as the conditions of this test could not be fulfilled (>20% of the cells has an expected count <5)

Among the patients who fulfilled the criteria for GCT based on their self-reported data on family history and information in their medical record (e.g., age at diagnosis, and characteristics of the tumor (*n* = 33)), 79% reported they were aware of the possibility to obtain GCT (83%; *n* = 19 of 23 Moroccan and 70%; *n* = 7 of 10 Turkish patients) and 64% of the patients eligible for GCT reported that they had undergone GCT (61% (*n* = 14) among 23 Moroccan, and 70% (*n* = 7) among 10 Turkish patients). Additionally, three Moroccan patients had received GCT despite, to our knowledge, not fulfilling the criteria for GCT.

#### Possible explanations for lower participation: perceptions of patients and healthcare professionals

The interviews and focus groups among the Turkish and Moroccan patients revealed several barriers as well as motives for participation in GCT. The themes that originated from the patient data are described in the first column of Table 3. In the second column of the table, the themes that emerged from the focus groups among healthcare professionals are presented.

**Table 3:** Possible motives and barriers related to participating in GCT

Perspective of patients^a^	Perspective of healthcare professionals^b^
Barriers	
Cultural factors	
Cancer taboo, cancer as a death sentence	Cancer taboo, more secrecy
	Lack of familiarity of healthcare professional with migrant culture of patients
Limited knowledge of family cancer history	Limited knowledge of family cancer history
Cancer as an unknown disease	More difficulties in accessing family history
Limited information given to children (in country of origin), not informed when going abroad	
Fewer close relationships with second degree family members (Moroccan women)	
Nondisclosure to family members to spare them grief (cancer taboo)	Nondisclosure wish of cancer diagnosis by family members
Psychosocial factors	
Lacking social support, disagreement of family members, especially daughters, to obtain GCT	
Anxiety of the patient	Different mind-set
Patient-physician communication	
Language difficulties	Language barriers
	“Patient not a communication partner” (translator-physician)
	Relying on translator/“not sure translation is correct” (nurses)
Lack of familiarity with health care	Limited knowledge about breast cancer and health care in T/M patients
Limited knowledge about breast cancer and illness in general	Poorly educated; you have to teach the basics of health and diseases first
Difficulties in formulating the right questions	Other questions of Turkish/Moroccan patients
Being “numb” after disclosure of breast cancer diagnosis	
Afraid to ask questions	Fewer questions of Turkish/Moroccan patients
Too little time with surgeon	Consultations take more time, referral to GCT might be postponed/delayed and “forgotten”
Doctor’s role, faith is in their hands Doctor’s advice taken	Different contact with migrant patients; doctor is seen as the “healer”
Motives	
Preventive options for oneself	In general, positive attitude to GCT observed
Knowing whether family members, especially children, would be at risk; to gain reassurance	
Religious belief; according to Islam/Allah, you have a duty to investigate in order to become well	
Doctor’s advice taken	More assertiveness among younger patients
	Support of nurse practitioners referring patients to GCT
Making patients aware of the possibilities, “they should make GCT obligatory”	

^a^Based on data from both the interviews and the focus groups with Turkish and Moroccan breast cancer patients

^b^Based on the focus groups with medical professionals: surgeons, a radiation oncologist, and nurse practitioners

### Barriers for participation in GCT

#### Cultural factors

Some barriers are rooted in the values that characterize the society of origin. For generations, cancer was a taboo subject in the women’s countries of origin. Their conversations during the interviews showed that cancer is seen as a death sentence, which made it hard to talk about and impacted on family communication.

The cancer taboo was also recognized by the healthcare professionals (second column of Table 3). They perceived more secrecy among Turkish and Moroccan women about their cancer diagnosis. Moreover, the focus groups pointed towards cultural differences, for example, lack of familiarity of some of the healthcare professionals with migrant culture of patients. This cultural gap might be a barrier for patient-physician communication but also for the recognition of certain characteristics such as correct estimation of the patient’s age.

The interviews and focus groups for patients showed that there is limited knowledge of family cancer history among Turkish and Moroccan women. While living in the country of origin, (breast) cancer seemed to be less known or even unknown and was therefore rarely discussed. Cancer was called “the evil disease” and was considered taboo. A contributing factor to this was that, in the villages where the majority of the women lived previously, visits to the doctor were scarce, and cancer remained therefore unknown. When finally diagnosed, it almost always meant a death sentence. The social norm was that cancer was not a subject to be discussed, especially not with children. The women who migrated seemed to have very little information about their family history of cancer, if they were aware of the existence of cancer at all (see quotes 1 and 2).

- Quote 1, INT74, Turkish, aged 55 years at breast cancer (BC) diagnosis

*At the time, cancer didn’t exist yet. At least, we weren’t familiar with the name. There was no knowledge and no research was conducted. It was just not mentioned. Only 38 years later when my ex-husband’s uncle was taken to the hospital in Ankara and died of cancer in his small intestines, we heard the word ‘cancer’. You had ‘verem’. The word ‘cancer’ wasn’t known at the time*.

- Quote 2, INT75, Moroccan, aged 50 years at BC diagnosis

*My father suffered from cancer too, only it was never mentioned. The disease ‘cancer’ was a totally unknown disease. Nobody knew what people died of. I only learned what cancer was all about when I came to the Netherlands. I saw it on TV. I had never heard of cancer although I was a grown-up woman when I came to the Netherlands*.

Limited knowledge of family history was mentioned as a barrier among healthcare professionals as well. The healthcare professionals reported difficulty in assessing family history among Moroccan and Turkish patients because these patients seemed to be less aware of their family history.

The participating nurses suggested that there was more secrecy about breast cancer among Moroccan and Turkish women, as if secrecy was a part of their culture. They referred to situations in which family members did not want the patient to know they had cancer.

Contrary to past customs and to the way of life in the country of origin, it now seems more natural for the migrant women living in the Netherlands to talk about their own breast cancer with closer family. A transition in this way of thinking and the resulting contradictions with their initial beliefs are shown in quotes 3 and 4.

- Quote 3, INT69, Moroccan, aged 53 at BC diagnosis

*In the old days, people didn’t talk about it. In Morocco, feelings of shame played a major role. My aunt in Morocco, for instance, had breast cancer. She knew because she had found a hard lump in her breast that was sticking out. She would not go and see a doctor as she felt embarrassed to undress in front of a physician; to show her bare breasts. [...] In my family there is no more shame about cancer. It is being discussed like a normal subject. At the time, when somebody had cancer, it was not discussed as we thought that the person would only have a few days left to live. This was caused by a lack of information. Many people in Morocco suffer from cancer while they don’t even know it. I have another aunt in Morocco who has cancer and everybody knows except for her, because her children won’t tell her. [....] Allegedly this is for the benefit of my aunt, as the stress would be additionally negative*.

- Quote 4, INT72, Turkish, aged 37 at BC diagnosis

*It is especially difficult because, in our culture, cancer is always associated with death, and that’s what I think is the hardest part. It is the first reaction of people. I’m not talking about my siblings. They are so close to it, they do understand. They also studied and have their own views on the matter. But uncles and aunts, who all live in Turkey, for them it’s a complete disaster, what’s happening to me. So, that’s really difficult for me*.

To demonstrate the extent of the communication about breast cancer within migrant families, the patients were asked to identify which family members were aware of the patient’s own breast cancer diagnosis. Most interviewees reported that they thought everybody in their family was aware of their diagnosis. Among Moroccan patients, members of the extended family were less likely to be aware of the patient’s breast cancer (62% reported everybody was aware) compared to Turkish family members (92%; see Table 1).

The closeness of relationships with second degree family members plays a role in informing them. When talking about breast cancer with more distant relatives such as nieces and aunts, some hesitation could still occur. Among Moroccan women, the extended family (including aunts and cousins) seems less close compared to Turkish families, and sometimes, the relationships are filled with distrust (see quote 5).

- Quote 5, INT23, Moroccan, aged 38 at BC diagnosis

*Within my family, everything is being discussed. Everything I feel and know about discovering breast cancer in an early stage, I share with my family. With other relatives, it’s different. Strangers will only say: “what a pity”, but close relatives, they are happy for me. You can even be bullied over something that Allah gave you. That is not very nice*.

Saving family members from worry was another reason for not informing them, which accounted mostly for the closer family members, such as parents or children. Some women did not want to tell close family members to spare them the grief of knowing their beloved one has cancer (quote 6). This might have to do with the cultural belief that cancer results in death, which makes it harder to bring up the topic.

- Quote 6, INT10, Moroccan, aged 46 at diagnosis

*I find it really difficult [emotionally]. I can’t tell my mum. She’s old and she’s ill herself. I don’t want to burden her with my disease. She might become even more ill. My brothers and sisters in Morocco don’t know either. That’s because I’m afraid my mum will find out through my brothers*.

### Psychosocial factors

Nonparticipation in GCT among those eligible and aware of the procedure had to do with the lack of social support (disagreement of family members) and the anxiety of the patient herself. The cancer taboo might play a role here as well.

Relatives involved in the patient’s care, especially daughters, seem to have a relatively large influence in the patient’s health-related decision-making, such as participation in GCT. Some of the patients argued that family members disagreed with undergoing GCT. Particularly among women who have language difficulties, daughters are very involved in the patient’s breast cancer diagnosis and treatment, and when they do not want to cooperate, they might have more influence than among non-Turkish or Moroccan patients. To put this into perspective, we would also like to mention the example of a young woman who made an autonomous decision to undergo GCT despite the reluctance of her brothers.

Anxiety about breast cancer seems to prevent some of the patients from seeking a referral for cancer GCT. Knowing that their hereditary cancer will have an impact on the breast cancer risk for themselves and their relatives worries them about the outcomes of GCT, and they do not want to be confronted with that.

The healthcare professionals noticed a difference in the mind-set of patients. Women from Dutch origin are usually aware of the possible side effects of cancer treatment and look beyond survival, whereas many Turkish and Moroccan women focus primarily on survival. This might have to do with the fact that in the Turkish and Moroccan communities, much emphasis is placed on the deadliness of cancer. This is perhaps reflected in their state of mind or attitude during the patient-physician communication.

### Patient-physician communication

Other barriers to referral to GCT lie within the patient-physician communication. All patients had consulted a surgeon in one of the six participating hospitals. Although the patient satisfaction with the care and the information given by the surgeon was high (respectively, 89.7 and 87.2% reported to be (very) satisfied; see Table 4), 68.8% of the patients reported that they did not always fully understand what the doctor had told them.

**Table 4:** Patient-surgeon communication

	Moroccan *N* = 52	Turkish *N* = 26	Total *N* = 78	*p* value
(Very) satisfied with care of surgeon	90.4% (47)	88.5% (23)	89.7% (70)	NS
(Very) satisfied with information provided by surgeon	86.5% (45)	88.4% (23)	87.2% (68)	NS
Having difficulties understanding surgeon^a^	70.6% (36)	65.4% (17)	68.8% (53)	NS
One or more visits being translated^b^	71.9% (23/35)	82.6% (19/23)	76.4% (42/55)	NS
Had been offered professional translator^b^	9.7% (3/31)	30.0% (6/20)	17.7% (9/51)	–
Want professional translator^b^	25.8% (8/31)	35.0% (7/20)	29.4% (15/51)	NS
Trust Dutch doctors most Trust Turkish/Moroccan doctors most Same	38.8% (19) 4.1% (2) 57.1% (28)	27.3% (6) 9.1% (2) 63.6% (14)	35.2% (25) 5.6% (4) 59.2% (42)	–

*NS* not significant

^a^Having a translator is recorded as having difficulties in understanding the surgeon

^b^Selection of the women who were (partly) interviewed in the language of origin

Many women had difficulties in processing the information from the surgeon (see Tables 3 and 4 and quotes 7–9). Besides the language difficulties, the thematic analysis showed several factors that might prohibit the migrant breast cancer patients from becoming fully informed, such as “lack of familiarity with health care”, and “limited knowledge about breast cancer and illness in general”. This might prohibit them from asking the right questions. Furthermore, the disclosure of the breast cancer diagnosis seems to keep them in a state of apathy, impeding them from asking questions (see quote 10).

- Quote 7, INT21, Moroccan, aged 41 at BC diagnosis

*If there were things that I didn’t understand, I would ask for plain examples and then I would know. And sometimes I didn’t understand and would just leave it that way*.

- Quote 8, INT12, Moroccan, aged 38 at BC diagnosis

*You’re just not used to it; it’s about your body. Sometimes you’re told things by a doctor and you go like, yeah, whatever*.

- Quote 9, respondent focus group

*Because it’s hard for us to express ourselves, we don’t get enough attention from our GP, nor from the hospitals. They expect a lot from you. While you don’t even know so much about the disease. If you did know all about it, you wouldn’t go and see a doctor every time*.

- Quote 10, INT43, Moroccan, aged 37 at BC diagnosis

*The information was sufficient, but at the time it didn’t land (I didn’t realise). I felt numb. I saw his lips moving, but I didn’t hear what he said. I was like a puppet. The next day I called my GP and he called the hospital and then gave me more information*.

Moreover, there might be a resistance to ask questions because they suspect the answers might have a negative consequence (quote 11). Some patients feel there is lack of time to ask questions to the surgeon. It is also possible that the doctor is sometimes placed on a pedestal (see quote 12). In their land of origin, doctors are high up in the social hierarchy. This position of the doctor may well prohibit the patients from asking further questions.

- Quote 11, INT74, Turkish, aged 55 at BC diagnosis

*I deliberately didn’t ask in order to avoid more sadness, insecurity and fear in my life. That’s why I didn’t go into detail. Patiently, I did what the doctors told me to do*.

- Quote 12, INT 32, Turkish, aged 48 at BC diagnosis

*She was a very good surgeon. Dr xxx is such a sweet doctor. You know, I can almost say that I overcame the disease thanks to my surgeon! Every time I went there, she welcomed me with a smile and made me forget all about my disease. She’s a really nice, sweet and funny lady. I will do doea for her, may Allah give her all she desires*.

Similarly, the healthcare professionals also consider language differences to be an important barrier. In cases where an interpreter is present, the patient is often not a “communication partner”; the conversation about the patient takes place with the family translator. Thus, the migrant patient is often not directly involved in the communication, because the conversation is between the children/husband/interpreter and the doctor.

Nurses agreed that there were many language difficulties and they were not sure if everything was translated correctly. Occasionally, and more so in the past, the family member who was translating (on most occasions the husband or a son/daughter) did not want the patient to know she had breast cancer. It was difficult to know how to deal with that because the medical staff wanted to respect the patient’s culture as well.

Besides language problems and cultural differences (e.g., social norms about cancer), medical staff also noticed differences in health education. The physicians have to educate many women about basic aspects of the body and the health system. It was observed that non-Turkish or Moroccan women were asking more frequent and different kinds of questions, for example, about side effects of chemotherapy or about the risks for their children to develop cancer. The Moroccan and Turkish women seemed to be less familiar with breast cancer and had a poorer level of education in general.

According to the healthcare professionals, especially the surgeons, a consultation with Moroccan and Turkish women takes more time due to the educational differences and lack of familiarity with health in general. As a result, it is possible that the subject of GCT is postponed. When postponed, it could be more likely that the subject is forgotten to be brought up at a later date.

Doctors feel they have a different role as “healer” with migrant patients compared to non-migrant patients, and that, the former look up to the doctor. This seems to influence their interaction with the patient (see quotes 13 and 14).

- Quote 13, focus group, surgeon

*Well, let me put it like this, I’ve always felt that as a doctor, you are … you have the role of a magician. Because many people have a tendency to trust you immediately and completely and that you take care of them and they want to hear they’re doing well, or that it’s not so bad. So you do have, you know, a different role as a doctor, I think, than with the native group, the Dutch group. In a way, you’re more... I’d call it a magician, you’re assigned a role like that. What you say is imperative. Whilst the native group, they’re more critical and often say, ‘Well ... is that right?’*.

- Quote 14, fragment from the focus group of nurse practitioners

*R1: I remember when one of our surgeons left and there were some of those foreign women who really adored him and went, “Oh doctor…” They cried because he left. Apparently, they really become attached to a person. And that’s quite exceptional […].*

*R1: Yeah, like somebody helps me to survive, so to speak.*

*R3: Yes, but imagine you wind up in a foreign hospital, where you don’t know the language. Well, you’d cling to anyone who’s being nice to you.*

*R1: Yes, that’s true. Just like with this category, where sometimes you can’t grasp it all, and it’s all the more important to be very nice to them.*

*R3: Yeah, well, I recognize that.*

#### Motives for participation in GCT

Several factors seem to stimulate participation in GCT, such as the preventive options for oneself and the fact that information is obtained about children and sisters’ breast cancer risks.

Although some women perceived their breast cancer diagnosis as a test of God, religious beliefs do not seem to prohibit the women in our sample from obtaining GCT. Rather, it could be seen as a stimulus. When questioned in the focus groups, the women suggested that because Allah gave them the disease, they had a duty to investigate in order to recover, which might actually motivate women to pursue GCT (see quote 15). Another trigger to undergo GCT was a doctor who told them to do so. The importance of awareness of the possibilities of GCT among all women and even making GCT obligatory were mentioned as well (see Table 3).

- Quote 15, fragment from the focus group of Turkish patients

*S4: From the point of view of Islam, it is important to look for opportunities [about genetic counseling]. You may say, ‘Well, I’m ill. I’ll do nothing. I won’t undergo examinations and testing’. The Islam faith tells that you have to get examined. There is a saying in Turkish ...*

*S6: You should not stay put, but you must continue to investigate*.

Although many barriers might prohibit participation in GCT directly or indirectly, the healthcare professionals believe that there seems to be turning point. When compared with diagnoses from 20 years ago, Turkish and Moroccan patients, especially the younger women, have become more assertive and breast cancer seems to be less of a taboo. In general, when asked directly about the possibilities of GCT, there seems to be a positive attitude towards it.

In the focus group of healthcare professionals, a positive “task” in the checkup for referral was seen to by nurse practitioners. The limited time of the physician was considered a barrier to referring GCT as it might be overlooked; however, the reminders of nurse practitioners were found to be very useful, according to the physicians.

## Discussion

Our study showed that half of the total group of patients was interviewed, and nearly 80% of eligible patients were aware of GCT. Of these, approximately 60% of eligible patients had actually obtained GCT. Moroccan and Turkish patients themselves, as well as the healthcare professionals, identified significant language and health literacy difficulties. Among these patients are mainly first-generation migrants with a low level of education; these difficulties were observed in the patient-doctor communication. This made it harder for them to fully access genetic counseling and testing. Moreover, cultural factors (e.g., social norms and customs), limited knowledge of the family cancer history, and anxiety about cancer were determinants of nonparticipation.

The first step to participation in GCT is having access to a provider (Forman and Hall 2009). In our study, all respondents were breast cancer patients and thus had access to a physician that could refer them to GCT. In the Netherlands, physicians are according to the Medical Treatment Act (WGBO) obliged to inform patients fully about their diagnosis, treatment, and prognosis. Geographic and economic barriers to GCT as reported by others (Forman and Hall 2009) are less likely in a small and densely populated country such as the Netherlands, where the costs of genetic counseling and DNA testing are covered by health insurance with the exception of the obligatory deductible excess of minimally 385 euros per year, which may or may not have already been used for a particular patient. Similar barriers to GCT as mentioned by Forman and Hall (2009) found among Turkish and Moroccan patients had to do with awareness of GCT, risk awareness of having hereditary cancer, and language and cultural factors. Our data shows that the cancer taboo as a cultural factor is still present in Turkish and Moroccan communities. It hinders the inter-family communication about the family cancer history, resulting in an inaccurate risk awareness by the both the patient and the physician. These findings agree with others who have studied barriers to GCT among other ethnic groups (Sussner et al. 2009; Vadaparampil et al. 2006); acculturation seems to play a role in the referral process among Turkish and Moroccan women. We have shown that awareness of the possibility to obtain GCT was associated with variables relating to acculturation, reflecting part of the language problems. Patient-doctor communication is limited by language and cultural barriers (e.g., attitude towards physician), hindering referral to GCT.

The health care system in the Netherlands is built upon increased patient assertiveness. This is also the case for GCT, where patients are usually referred by their physician (surgeon or general practitioner), but the initiative to undergo GCT may also come from the patient who, for example, asks questions to the doctor because of her family cancer history (van Riel et al. 2012). The limited knowledge of family cancer history among Turkish and Moroccan patients prevents them from voicing their concerns. Furthermore, the limited awareness of GCT makes it impossible for some of the women to ask about the possibility of obtaining GCT. Moreover, our results suggest that a lower level of health literacy, defined as “the degree to which individuals can obtain, process, understand, and communicate about health-related information needed to make informed health decisions” (Berkman et al. 2010), is an important determinant of the lower participation rate of Moroccan and Turkish breast cancer patients. The cultural background of the women, especially their experiences with doctors, their limited knowledge of cancer, and language difficulties, may influence the way in which individuals interact with clinicians, the health care system, and their health-related goals as stated by Berkman et al. (2010). In their country of origin, doctors are high in the social hierarchy. Also, in the Netherlands, doctors are usually highly revered by migrant women; they put their faith in the hands of the physician. In this way, these women can remain sub-assertive and obedient, instead of taking an assertive role.

Although others (Forman and Hall 2009; Thompson et al. 2003) found that an expressed belief in divine order or purpose was more common among minorities and hypothesized that spirituality may also mold behaviors related to genetic risk and cancer prevention, we found that religion could also be a motivating factor to participate in GCT. Because breast cancer is considered a test, it is the women’s duty to grasp every possibility to become well (Thompson et al. 2003). Knowing one’s own risk and that of the family members was an important motivation to obtain GCT as well. Our study showed that there are several personal motives to obtain GCT when potential barriers have been overcome.

### Limitations

The non-response analysis showed that the respondents were younger than the non-respondents, and one of the reasons for rejecting the telephone interview was that the patients did not want to be reminded of the period of time when they had breast cancer. Although we had interviewees who argued that it was difficult for them to talk about breast cancer, our sample might be biased towards those for whom breast cancer was less of a taboo and those who were more familiar with GCT. The telephone interviews were done by a research team of four bilingual research assistants. Having different individuals taking interviews risks leading to variation in the approach and follow-up questions. By supporting them closely (initial training, reflection on results, and personal follow-up meetings), we tried to minimize the possibility of information bias.

### Practice implications

Our study showed differences between Turkish and Moroccan patients in disclosing the patient’s breast cancer to family members. Distant members of Moroccan families such as aunts and uncles were less likely to be aware. This should be kept in mind when accessing family cancer history. Because of the difficulties in disclosing the breast cancer diagnosis to family members, healthcare professionals could be trained in how patients could learn skills to disclose “bad news” information to their family members, in this way helping them to spread health education messages and to enhance genetic counseling.

In case of a moderately or highly increased breast cancer risk, periodic surveillance of those with an increased risk is recommended and/or preventive surgery for carriers of a BRCA1/2 gene mutation (Chen and Parmigiani 2007). To minimalize health disparities, education about the process of referral to GCT might help to empower migrant women to ask questions about the procedure and to enhance shared decision-making. Moreover, physicians should be aware of their limited communication capabilities, as well as interaction with migrant patients. Future studies should focus on the possibility of personalized training for physicians on the discussion and awareness of cancer genetic counseling with migrant breast cancer patients in order to guarantee equal access to breast genetic counseling for all.
